# The association of dietary inflammatory index with urinary risk factors of kidney stones formation in men with nephrolithiasis

**DOI:** 10.1186/s13104-020-05206-y

**Published:** 2020-08-08

**Authors:** Niloofarsadat Maddahi, Habib Yarizadeh, Seyed Mohammad Kazem Aghamir, Shahab Alizadeh, Mir Saeed Yekaninejad, Khadijeh Mirzaei

**Affiliations:** 1 Department of Community Nutrition, School of Nutritional Sciences and Dietetics, Tehran University of Medical Sciences (TUMS), Tehran, P.O. Box: 14155-6117, Iran; 2 Department of Urology, Sina Hospital, Tehran University of Medical Sciences, Tehran, Iran; 3 Department of Epidemiology and Biostatistics, School of Public Health, Tehran University of Medical Sciences, Tehran, Iran

**Keywords:** Dietary inflammatory index, Kidney stones, Hypercalciuria, Hypocitraturia, Hyperoxaluria, Hyperuricosuria, Hypercreatinuria

## Abstract

**Objective:**

Inflammation plays a leading role in the pathogenesis of nephrolithiasis. The association of the dietary inflammatory index (DII) with urinary lithogenic factors is unclear. This study aimed to evaluate the relation of DII to urinary risk factors of kidney stones formation.

**Results:**

Of 264 participants, 61.4% (n = 162), 72% (n = 190), 74.6% (n = 197), 68.6% (n = 181), and 80.3% (n = 212) had hyperoxaluria, hypercreatininuria, hypercalciuria, hyperuricosuria, hypocitraturia, respectively. There was a significant increasing trajectory in urinary calcium, uric acid, and creatinine as well as a decreasing trend in urinary citrate across tertiles of DII score (all P = ≤0.001). After multivariate adjustment for energy intake, age, physical activity and body mass index, high DII scores were associated with elevated odds of having hypercreatininuria (OR = 2.80, 95%CI: 1.10–7.12, P_trend_ = 0.04), hypercalciuria (OR = 7.44, 95%CI: 2.62–21.14, P_trend_ ≤ 0.001), hyperuricosuria (OR = 2.22, 95%CI: 1.001–4.95, P_trend_ = 0.05), and hypocitraturia (OR = 5.84, 95%CI: 2.14–15.91, P_trend_ ≤ 0.001). No association was identified between DII and hyperoxaluria.

## Introduction

Nephrolithiasis is one of the most prevalent urologic disorders and impose a substantial burden on human health globally [1]. The high recurrence rate of kidney stones is yet unsolved [2, 3].Thus, there is an urgent need to target modifiable risk factors to prevent the development and recurrence of renal stones.

Higher urinary excretions of oxalate, calcium, creatinine, and uric acid as well as lower excretions of citrate are potential modifiable urinary lithogenic risk factors involved in the formation of kidney stones [4–6]. Inflammation is also another mechanism which plays a leading role in the pathogenesis of nephrolithiasis [7]. Dietary modifications toward decreasing inflammation may have a potential to prevent kidney stones or their recurrence. Several micronutrients or foods such as magnesium, vitamin E, vitamin C, carotenoids, fruits, and fish had an anti-inflammatory impact [8–11]. In contrast, simple sugars, red meats, high-fat dairy products, and refined grains are associated with elevated inflammatory markers [12]. Nevertheless, nutrients or foods are not consumed separately but as part of the whole diet [13–15]. The Dietary Inflammatory Index (DII) is developed to measures the overall inflammatory potential of diets [16], which has been recognized to be related to the biomarkers of inflammation [17]. A proinflammatory diet has been found to be related to the reduced kidney function [18]. However, there is no study investigating the relation of DII to urinary lithogenic factors. Therefore, this study aimed to assess the association of DII with hypercalciuria, hypocitraturia, hyperoxaluria, hyperuricosuria, and hypercreatinuria in patients with nephrolithiasis.

## Main text

### Methods

#### Subjects

This cross-sectional study was performed on a total of 264 stone former men (aged 18– 89 years) in Tehran, Iran in 2016. Participants were recruited from the Urology Research Center of Sina Hospital, Tehran, Iran. Inclusion criteria for this study were having a history of kind stone formation and age ≥ 18 years. People with a history of, thyroid disease, fatty liver disease, malignancy, stroke, diabetes, cardiovascular disease, and hypertension were excluded. Participants who were on medications such as corticosteroids, diuretics, anti-cancer drugs, multivitamins, potassium citrate, calcium, and vitamin D or C supplements were not eligible for this study. Furthermore, all alcohol drinkers and drugs abusers were excluded. Patients were included in the study after signing written informed consents.

#### Dietary assessment

Usual food intake of patients during the previous year was measured by a validated semi-quantitative 168-item food frequency questionnaire) FFQ([19]. DII was calculated using the method reported by Shivappa et al. [20]. The DII is based on 1943 scientific papers scoring 45 food parameters based on whether they elevated (+1), reduced (−1) or had no impact (0) on six inflammatory biomarkers [C-reactive protein, interleukin (IL)-1 beta, IL-10, IL-4, IL-6, and tumor necrosis factor-alpha). As mentioned, Shivappa et al. calculated DII according to the 45 food parameters. As dietary patterns of different populations are different with each other, some food parameters used in the study by Shivappa may not be available in different FFQs. Hence, researchers calculate DII according to available foods in the FFQ by modification of the method used by Shivappa. et al. [21]. In the current study, the DII score was calculated using the corresponding 32 food parameters available from the FFQ used in our study. This approach has been used broadly in the previous studies [21]. The DII score was calculated with the use of the corresponding 32 nutrients or food parameters available from the FFQ, including energy, protein, total fat, carbohydrate, dietary fiber, mono-unsaturated fatty acids, n-3 fatty acids, n-6 fatty acids, poly-unsaturated fatty acids, saturated fatty acids, cholesterol, trans fatty acids, vitamin A, thiamin, niacin, riboflavin, Vitamin B-6, folate, vitamin B-12, vitamin E, vitamin C, Vitamin D, b-carotene, iron, magnesium, zinc, selenium as well as caffeine, onion, green/black tea, paper, and garlic. The inflammatory effect scores for dietary components used for calculation of DII in this study are reported in Additional file 1: Table S1. To calculate the DII score for each participant, the mean intake of each nutrient or food parameter was standardized by subtracting mean global intake of food items from the actual individual’s intake and dividing it by the global SD to create a z-score. *Z*-score is used to express an individual’s exposure relative to the standard global exposure. This approach both anchors the individual’s exposure to a robust range of dietary patterns in a variety of cultural traditions and obviates completely the problem of non-comparability of units because the *Z*-scores is independent of the units of measurement. These z-scores then were converted to proportions and centered by multiplying values by 2 and subtracting 1 to normalize the scoring system and to avoid skewness. The centered percentile values for food items were then multiplied by the corresponding food item-specific inflammatory effect scores to obtain the food item-specific DII scores. Calculation of DII for carbohydrate intake in a participant in our study as an example for DII calculation is presented in Additional file 1: Table S2; similar approach was followed for the calculation of DII for other nutrients. Information about global daily mean intake, standard deviation for global intake, and overall inflammatory effect score of all nutrients/food items used for DII calculation is reported in the study by Shivappa et al. [20]. The overall DII score for each individual was calculated by summing food item-specific DII scores [20]. Higher DII scores indicate a more pro-inflammatory diet, while lower DII scores indicate a more anti-inflammatory diet.

#### Measurements of study outcomes

The 24-h urine samples were collected from all participants and urine was analyzed using an AutoAnalyzer as described previously [22]. Hyperoxaluria was defined as the urinary oxalate ˃ 40 mg/day, hypocitraturia as urinary citrate of < 450 mg/day, hyperuricosuria as urinary uric acid over 0.8 g/day, hypercreatininuria as urinary creatinine of ˃ 24 mg/day, and hypercalciuria as a urinary calcium ≥ 250 mg/day [22].

#### Measurement of other variables

General Information was obtained using interview. Physical activity was measured using of the International Physical Activity Questionnaires (IPAQ) [23]. Body weight was measured in minimal clothing after removal of shoes by a digital scale (Seca, Germany) with a precision about 0.1 kg. Height of individuals was assessed in standing position, without shoes, using a calibrated stadiometer (Seca, Germany) to the nearest 0.1 cm. BMI was calculated as weight divided by the square of height (kg/m2).

#### Statistical analyses

DII was categorized into tertiles: T1 (−3.72 to −0.74); T2 (−0.73 to 0.92); T3 (0.93 to 3.99). Analysis of variance (ANOVA) and Chi square tests were used to compare continuous and nominal/ordinal variables across tertiles of DII, respectively. Continuous variables are reported as mean ± SE and nominal/ordinal variables as frequency. Odds ratio (OR) and 95% confidence interval (CI) for the relation of DII to study outcomes was calculated using the logistic regression analysis. Statistical significance was set at *p *≤ 0.05 for all tests. All analyses were undertaken using the statistical Package for Social Science (Version 22.0; SPSS Inc., Chicago IL, USA).

### Results

Participants in the highest tertile of the DII had significantly higher total daily energy intake (P = ≤ 0.001) and lower physical activity (P = 0.01) than those in the other tertiles. There was a significant increasing trajectory in urinary calcium (P = ≤ 0.001), uric acid (P = ≤ 0.001), and creatinine (P = ≤ 0.001) and a decreasing trend in urinary citrate (P = ≤ 0.001) across tertiles of DII score (Table 1).

**Table 1:** Characteristics of the study participants across tertiles of the DII score

	Total	Dietary inflammatory index score			
	N = 264	Tertile 1 (n = 88)	Tertile 2 (n = 88)	Tertile 3 (n = 88)	*P* value
Age (year)	42.68 ± 0.78	41.58 ± 1.31	43.83 ± 1.38	42.64 ± 1.37	0.50
Height (cm)	173.52 ± 0.47	173.18 ± 0.78	173.56 ± 0.86	173.81 ± 0.84	0.86
Weight (kg)	80.73 ± 0.86	80.93 ± 1.60	81.36 ± 1.51	79.92 ± 1.39	0.78
BMI (kg/m2)	26.75 ± 0.24	26.94 ± 0.47	26.93 ± 0.42	26.37 ± 0.37	0.55
Physical activity score	4851 ± 453	6654 ± 991	3413 ± 669	4486 ± 611	0.01
Energy intake (kcal/day)	2435.86 ± 35.44	2205.91 ± 44.04	2380.07 ± 59.24	2721.61 ± 66.05	≤ 0.001
Urinary creatinine/kg weight (mg/day)/kg	26.50 ± 0.49	24.48 ± 0.87	25.51 ± 0.92	29.51 ± 0.63	≤ 0.001
Urinary citrate (mg/day)	350.94 ± 9.69	394.30 ± 18.43	358.42 ± 17.25	300.10 ± 12.74	≤ 0.001
Urinary oxalate (mg/day)	45.99 ± 1.18	44.73 ± 2.12	48.72 ± 2.13	44.52 ± 1.88	0.26
Urinary uric acid (g/day)	857.61 ± 17.09	789.06 ± 32.06	823.79 ± 28.21	959.98 ± 25.28	≤ 0.001
Urinary calcium (mg/day)	329.02 ± 8.02	288.25 ± 12.74	325.11 ± 15.52	373.70 ± 11.74	≤ 0.001
Job status					0.22
Engineer/physician	12	2	5	5	
Clerk	57	20	19	18	
Student	4	2	0	2	
Teacher	4	2	0	2	
Self-employed	77	28	28	21	
Retired	47	14	19	14	
Worker	60	20	14	26	
Unemployed	3	0	3	0	
Marital status					0.82
Married	241	81	81	79	
Single	23	7	7	9	
Education level					0.68
Illiterate	10	3	4	3	
≤ Diploma	190	59	66	65	
University degree	64	26	18	20	

Categorical variables are presented as frequency (n), and continuous variables as mean ± S.E. One-way ANOVA was used for continuous variables and person’s Chi square test for categorical variables

#### DII score and urinary lithogenic factors

In the crude model, it was found that higher adherence to the DII was significantly related to the increased odds of hypercreatininuria (OR = 7.60, 95%CI: 3.28−17.61, P_trend_ ≤ 0.001), hypercalciuria (OR = 10.45, 95%CI: 3.84−28.39, P_trend_ ≤ 0.001), hyperuricosuria (OR = 4.40, 95%CI: 2.16-8.94, P_trend_ ≤ 0.001), and hypocitraturia (OR = 6.04, 95%CI: 2.35−15.55, P_trend_ ≤ 0.001). After multivariate adjustment for energy intake, age, physical activity and BMI, high DII scores were associated with elevated odds of having hypercreatininuria (OR = 2.80, 95%CI: 1.10−7.12, P_trend_ = 0.04), hypercalciuria (OR = 7.44, 95%CI: 2.62−21.14, P_trend_ ≤ 0.001), hyperuricosuria (OR = 2.22, 95%CI: 1.001−4.95, P_trend_ = 0.05), and hypocitraturia (OR = 5.84, 95%CI: 2.14−15.91, P_trend_ ≤ 0.001). The relation of DII to hypercreatininuria, hyperoxaluria, and hyperuricosuria was not significant after adjustment for carbohydrate, fiber and protein intake (Table 2).

**Table 2:** Univariate and multivariate logistic regression models for the relation of DII score to urinary risk factors of kidney stone formation

	Dietary inflammatory index score			
	Model 1 (Crude model)	Model 2	Model 3	Model 4
	Odds ratio (95% CI)	Odds ratio (95% CI)	Odds ratio (95% CI)	Odds ratio (95% CI)
Hypercreatininuria				
T1	1	1	1	1
T2	1.62 (0.88–3.01)	0.92 (0.45–1.89)	0.85 (0.41–1.78)	0.84 (0.38–1.85)
T3	7.60 (3.28–17.61)	3.10 (1.23–7.81)	2.80 (1.10–7.12)	2.21 (0.78–6.28)
P value for trend	≤ 0.001	0.02	0.04	0.19
Hypocitraturia				
T1	1	1	1	1
T2	1.60 (0.81–3.17)	1.53 (0.75–3.11)	1.69 (0.82–3.49)	1.16 (0.55–2.44)
T3	6.04 (2.35–15.55)	5.64 (2.10–15.15)	5.84 (2.14–15.91)	3.40 (1.17–9.86)
P value for trend	≤ 0.001	0.001	≤ 0.001	0.03
Hyperoxaluria				
T1	1	1	1	1
T2	1.34 (0.72–2.48)	1.47 (0.77–2.79)	1.63 (0.84–3.16)	1.46 (0.74–2.88)
T3	0.86 (0.47–1.58)	0.99 (0.52–1.90)	1.12 (0.57–2.19)	0.87 (0.41–1.83)
P value for trend	0.64	0.95	0.78	0.69
Hyperuricosuria				
T1	1	1	1	1
T2	1.69 (0.92–3.12)	1.08 (0.55–2.13)	1.20 (0.59–2.42)	0.94 (0.45–1.95)
T3	4.40 (2.16–8.94)	2.14 (0.98–4.67)	2.22 (1.001–4.95)	1.35 (0.56–3.24)
P value for trend	≤ 0.001	0.06	0.05	0.53
Hypercalciuria				
T1	1	1	1	1
T2	1.34 (0.72–2.51)	1.13 (0.59–2.18)	1.02 (0.52–2.00)	0.96 (0.48–1.91)
T3	10.45 (3.84–28.39)	8.11 (2.88–22.83)	7.44 (2.62–21.14)	6.22 (2.06–18.77)
P value for trend	≤ 0.001	≤ 0.001	≤ 0.001	0.003

Model 2: adjusted for energy intake

Model 3: additionally adjusted for age, BMI, and physical activity

Model 4: adjusted for carbohydrate, fiber and protein intake

We also evaluated the association of dietary protein and fiber intake on urinary risk factors (Table 3). After adjustment for covariates, dietary intakes of protein and fiber were slightly related to the decreased odds of hypocitraturia (Protein OR = 0.96, 95%CI: 0.93−0.98; fiber OR = 0.96, 95%CI: 0.94−0.98) and hypercalciuria (Protein OR = 0.97, 95%CI: 0.95−0.99; fiber OR = 0.97, 95%CI: 0.95−0.99), while the intake of protein and fiber was not to associated with hypercreatininuria, hyperoxaluria, and hyperuricosuria.

**Table 3:** Multivariate logistic regression models for the relation of dietary fiber and protein intake, as continues variable, to urinary risk factors of kidney stone formation

	Fiber		Protein	
	Odds ratio (95% CI)		Odds ratio (95% CI)	
	Model 1	Model 2	Model 1	Model 2
Hypercreatininuria	0.98 (0.96–1.01)	0.98 (0.96–1.01)	0.98 (0.95–1.008)	0.98 (0.96–1.01)
Hypocitraturia	0.96 (0.94–0.98)	0.96 (0.94–0.98)	0.96 (0.93–0.98)	0.96 (0.93–0.98)
Hyperoxaluria	0.98 (0.96–1.006)	0.98 (0.96–1.005)	1.00 (0.98–1.02)	1.00 (0.98–1.02)
Hyperuricosuria	0.98 (0.96–1.00)	0.98 (0.96–1.002)	0.98 (0.96–1.008)	0.98 (0.95–1.006
Hypercalciuria	0.97 (0.95–0.99)	0.97 (0.95–0.99)	0.97 (0.94–0.99)	0.97 (0.95–0.99)

Model 1: adjusted for energy intake

Model 2: additionally adjusted for age, BMI, and physical activity

### Discussion

We revealed that, in stone former men, a diet with a high DII is significantly related to the increased odds of having hypercreatininuria, hypercalciuria, hyperuricosuria, and hypocitraturia, but not to hyperoxaluria.

It has been confirmed that kidney stone formers could be susceptible to recurrence in stones formation because of unhealthy dietary patterns [24]. Inconsistent with our finding, a study did not report any significant difference in creatinine across tertiles of DII in subjects with chronic kidney disease [25]. A randomized controlled trial study by Noori et al. [26] on recurrent stone formers showed that a DASH diet, which in contrast to a diet with a high DII, is featured by a high intake of whole grains, fruits, low-fat dairy products and vegetables, and a low intake of total fat, cholesterol, saturated fat, meat, and refined grains, is significantly associated with a decrease in calcium oxalate supersaturation and an increase in citrate excretion. Moreover, another study reported that greater adherence to the Mediterranean dietary pattern is related to the reduced risk for incident kidney stones [27]. The relationship between systemic inflammation and nephrolithiasis has been identified previously [7]. Since both DASH and Mediterranean diets attenuate inflammation [9, 28], the protective effects of these dietary patterns on kidney stones formation may be mediated, at least partly, by reducing systemic inflammation. A cross-sectional study conducted on diabetic patients also reported that higher intake of “vegetable and fish” dietary pattern is related to a lower creatinine rates [29]. Vegetables and fish, as components of DII, are identified to have anti-inflammatory effects [30, 31]. The DII is a tool to assess the overall impact of a diet on inflammatory potential [20], and is associated with markers of systemic inflammation including such as IL-6 [32], and CRP [17] [..]; IL-6 and CRP are two of the inflammatory biomarkers considered in the calculation of DII [20]. It has been revealed that the DII score is inversely related to the Dietary Approaches to Stop Hypertension Score (DASH), Mediterranean Diet Score, and Healthy Eating Index-2010 [33, 34]. Taken together, these findings support that a likely mechanism for the relation of DII scores to hypercreatininuria, hypercalciuria, hyperuricosuria, and hypocitraturia could be explained by the higher systemic inflammation level among people following a pro-inflammatory diet.

Since DII positively depends on protein intake that is also a metabolic risk factor for hypercalciuria, hyperuricosuria, and hypocitraturia, and on contrary, dietary fiber has a negative impact on DII and is a factor that can reduce calcium absorption, we also adjusted the analysis for protein, carbohydrate, and fiber intake to differentiate the metabolic impact of DII from its pro-inflammatory impact. It was found that DII is related to hypercalciuria and hypocitraturia independent of dietary intake of protein, carbohydrate, and fiber, however, the relationship between DII and hyperuricosuria disappeared, showing that this association may be resulted from metabolic effects of DII. Nevertheless, protein and fiber intake was inversely associated with hypercalciuria and hypercalciuria.

### Conclusion

In conclusion, this study found that a diet with high inflammatory property might be unfavorably associated with urinary risk factors of kidney stone formation in men with a history of nephrolithiasis.

## Limitation

First, since the participants of the current study were limited to men, our findings may not be generalizable to women; therefore, it is essential to conduct such a study on women too. Third, causation cannot be inferred the cross-sectional design of the present investigation. Finally, the calculation of DII by FFQ has a potential recall bias for the evaluation of dietary intake.

## Supplementary information

**Additional file 1: Table S1.** Inflammatory effect scores for dietary components used for calculation of DII. **Table S2.** calculation of DII for carbohydrate intake in a participant in our study as an example for total DII calculation.

## Data Availability

The data are not publicly available due to containing information that could compromise the privacy of research participants.

## Abbreviations

IPAQ: International physical activity questionnaires
DII: Dietary inflammatory index
PUFAs: Polyunsaturated fatty acids
CRP: C- reactive protein
FFQ: Food frequency questionnaire
ANOVA: Analysis of variance
OR: Odds ratio
CI: Confidence interval
BMI: Body mass index
