# Supplementary material for: The association of dietary inflammatory index with urinary risk factors of kidney stones formation in men with nephrolithiasis
Source: BMC Res Notes. 2020 Aug 8;13:373. doi: 10.1186/s13104-020-05206-y (PMC7414556; doi:10.1186/s13104-020-05206-y)
Supplement: Supplementary file 1 — Additional file 1: Table S1. Inflammatory effect scores for dietary components used for calculation of DII. Table S2. calculation of DII for carbohydrate intake in a participant in our study as an example for total DII calculation. [file 13104_2020_5206_MOESM1_ESM.doc]

Table S1. Inflammatory effect scores for dietary components used for calculation of DII

| Food parameters | Inflammatory effect score* |
| --- | --- |
| Energy (kcal) | 0.180 |
| Protein (g) | 0.021 |
| Carbohydrate (g) | 0.097 |
| Total fat (g) | 0.298 |
| Saturated fat (g) | 0.373 |
| Polyunsaturated fatty acids (g) | -0.337 |
| Monounsaturated fatty acids (g) | -0.009 |
| Omega-3 fatty acids (g) | -0.436 |
| Omega-6 fatty acids (g) | -0.159 |
| Cholesterol (mg) | 0.110 |
| Vitamin A (RE) | -0.401 |
| Carotene (μg) | -0.584 |
| Vitamin E (mg) | -0.419 |
| Vitamin B1 (mg) | -0.098 |
| Vitamin B2 (mg) | -0.068 |
| Niacin (mg) | -0.246 |
| Vitamin B6 (mg) | -0.365 |
| Vitamin B12 (mg) | 0.106 |
| Folic acid (mg) | -0.190 |
| Vitamin C (mg) | -0.424 |
| Magnesium (mg) | -0.484 |
| Iron (mg) | 0.032 |
| Zinc (mg) | -0.313 |
| Fiber (g) | -0.663 |
| Caffeine (g) | -0.110 |
| Saturated fatty acids (g) | 0.373 |
| Trans fatty acids (g) | 0.229 |
| Vitamin D (μg) | –0.446 |
| Selenium (mg) | –0.191 |
| Onion (g) | –0.301 |
| Green/black tea (g) | –0.536 |
| Paper (g) | –0.131 |
| Garlic (g) | –0.412 |

* A negative value indicates anti-inflammatory effect and a positive score indicates pro-inflammatory effect.

Table S2. calculation of DII for carbohydrate intake in a participant in our study as an example for total DII calculation.

| calculations | | | | Required data | | |
| --- | --- | --- | --- | --- | --- | --- |
| 4) calculation of DII for carbohydrate intake | 3) calculation of centered percentile value for carbohydrate intake | 2) z-score then were converted to proportion by fractional ranking of participants | 1) calculation of z-score for carbohydrate intake | Standard deviation for global carbohydrate intake | Global daily mean  intake of carbohydrate intake (gr) | Daily carbohydrate intake of subject (gr) |
| (centered percentile value for carbohydrate intake * overall inflammatory  effect score of carbohydrate (0.097))= 0.356 * 0.097 = **0.034** | ((proportion of carbohydrate intake * 2)-1)) =  ((0.6780* 2)-1))= 0.356 | Fractional rank for z-score of carbohydrate intake of this participant was 0.6780 | (Daily carbohydrate intake of subject - Global daily mean intake of carbohydrate intake) / Global standard deviation for carbohydrate intake = (336.86-272.2) /40 = 1.62 | 40.00 | 272.2 | 336.86 |
